# Comparison of Apical Extrusion of Debris by Using Single-File, Full-Sequence Rotary and Reciprocating Systems

**Published:** 2016-11

**Authors:** Maryam Ehsani, Robab Farhang, Azadeh Harandi, Saeid Tavanafar, Maryam Raoof, Saeedeh Galledar

**Affiliations:** 1 Associate Professor, Department of Endodontics, Faculty of Dentistry, Babol University of Medical Sciences, Babol, Iran; Dental Materials Research Center, Faculty of Dentistry, Babol University of Medical Sciences, Babol, Iran; 2 Assistant Professor, Department of Endodontics, Faculty of Dentistry, Ardabil University of Medical Sciences, Ardabil, Iran; 3 Assistant Professor, Dental Materials Research Center, Faculty of Dentistry, Babol University of Medical Sciences, Babol, Iran; Department of Endodontics, Faculty of Dentistry, Babol University of Medical Sciences, Babol, Iran; 4 Postgraduate Student, Department of Oral and Maxillofacial Surgery, School of Dentistry, Shiraz University of Medical Sciences, Shiraz, Iran; 5 Associate Professor, Department of Endodontics, Faculty of Dentistry, Endodontolgy Research Center, Kerman University of Medical Sciences, Kerman, Iran; Laboratory of Molecular Neuroscience, Neuroscience Research Center, Institute of Neuropharmacology, Kerman University of Medical Sciences, Kerman, Iran

**Keywords:** Dentistry, Endodontics, Root Canal Preparation, Instrumentation

## Abstract

**Objectives:**

During root canal preparation, apical extrusion of debris can cause inflammation, flare-ups, and delayed healing. Therefore, instrumentation techniques that cause the least extrusion of debris are desirable. This study aimed to compare apical extrusion of debris by five single-file, full-sequence rotary and reciprocating systems.

**Materials and Methods:**

One hundred twenty human mandibular premolars with similar root lengths, apical diameters, and canal curvatures were selected and randomly assigned to six groups (n=20): Reciproc R25 (25, 0.08), WaveOne Primary (25, 0.08), OneShape (25, 0.06), F360 (25, 0.04), Neoniti A1 (25, 0.08), and ProTaper Universal. Instrumentation of the root canals was performed in accordance with the manufacturers’ instructions. Each tooth's debris was collected in a pre-weighed vial. After drying the debris in an incubator, the mass was measured three times consecutively; the mean was then calculated. The preparation time by each system was also measured. For data analysis, one-way ANOVA and Games-Howell post hoc test were used.

**Results:**

The mean masses (±standard deviation) of the apical debris were as follows: 2.071±1.38mg (ProTaper Universal), 1.702±1.306mg (Neoniti A1), 1.295±0.839mg (OneShape), 1.109±0.676mg (WaveOne), 0.976±0.478mg (Reciproc) and 0.797±0.531mg (F360). Compared to ProTaper Universal, F360 generated significantly less debris (P=0.02). The ProTaper system required the longest preparation time (mean=88.6 seconds); the Reciproc (P=0.008), OneShape (P=0.006), and F360 (P=0.001) required significantly less time (P<0.05).

**Conclusions:**

All instruments caused extrusion of debris through the apex. The F360 produced significantly less debris than did the ProTaper Universal.

## INTRODUCTION

Complete root canal cleaning and shaping are necessary for successful endodontic treatment and periradicular healing. The aim of combining instrumentation and irrigation is to disinfect the root canal by removing microorganisms, pulp remnants and dentin chips, but debris may extrude through the apex into the periradicular tissues [1]. Confining the preparation to areas above the apical terminus can decrease the extrusion of debris into the periradicular tissues. Nevertheless, extrusion of even small amounts of debris can provoke postoperative inflammation and pain and delay the healing process [2]. Complications may include pain, swelling or both; these complications may necessitate emergency patient visits. A combination of pain and swelling is called flare-up [3]. The incidence of flare-ups is reported to be between 1.4% and 16% after 627 teeth with necrotic teeth were examined over a three-year period [4]. It seems that all current instrumentation techniques result in extrusion of intracanal content into the periradicular tissues, even when the area of preparation does not extend to the apical terminus, but the amount of extruded debris differs between instruments and file designs. Manual preparation is usually associated with more extrusion of debris compared to the use of nickel-titanium (NiTi) systems [5]. Recently, single-file, full-sequence NiTi systems (rotary and reciprocating) have attracted attention, and manufacturers have introduced new single-file systems with different kinematics and file designs. The Reciproc (VDW, Munich, Germany) and WaveOne (Dentsply Maillefer, Ballaigues, Switzerland) systems are made of a special heat-treated NiTi alloy called M-wire, which is claimed to increase flexibility and resistance to cyclic fatigue [6,7]. These systems use preprogrammed reciprocation motions that are specific to their file designs. The OneShape (Micro-Mega, Besanco, France), F360 (Komet Brassler, Lemgo, Germany) and Neoniti A1 (Neolix, Châtres-la-Forêt, France) are other single-file, full-sequence rotary NiTi instruments that are designed to prepare the entire root canal with only one instrument. They are made of traditional NiTi alloy and work in a continuous, clockwise, rotational motion.

The Neoniti A1 is produced with the electrical discharge machining method, which has advantages such as high precision, creation of various designs without tool constraints, and limited manufacturing stress to the file surface. This method also produces a rough surface, which can enhance the cutting abilities of the file [8]. No previous studies have compared these five single-file systems; thus, the aim of the present study was to compare the amounts of apically extruded debris by the five single-file systems and the ProTaper Universal (Dentsply Maillefer, Ballaigues, Switzerland) system, which was used as control.

## MATERIALS AND METHODS

### Sample collection:

The research protocol of this experimental study was approved by the Ethics Committee Board (Reference Number: 9440420). One hundred twenty human single rooted mandibular premolar teeth were used. The teeth were extracted for reasons unrelated to this study and used within two months of extraction. They were stored in 0.5% chloramine T (Merck, Darmstadt, Germany) for 48 hours and then transferred to distilled water at 4°C until they were used for experiments. Access cavity was prepared with diamond bur (Diatech, Coltene Whaledent, Altstetten, Switzerland), and the coronal portions of all canals were slightly flattened. A #10 stainless steel K-file (Dentsply Maillefer, Ballaigues, Switzerland) was used to negotiate the canal and to ensure canal patency and absence of obstruction. The working length (WL) was determined by subtracting 1mm from the visible file length. In addition, each tooth was radiographed from the meio-distal and buccolingual directions to confirm it had a single canal and to ensure absence of internal resorption and irregular anatomical structures. Any tooth with more than one root canal or apical foramen, an apical foramen larger than a #15 K-file (Dentsply Maillefer, Ballaigues, Switzerland), internal or external root resorption, or root canal curvature of more than 10° (measured by the Schneider’s method)[9] was replaced with a new tooth that met the inclusion criteria. Root surfaces of the samples were cleaned with a hand scaler and polished with pumice paste. The root lengths were measured from the cementoenamel junction, and one-way ANOVA performed in SPSS version 20.0 (SPSS Inc., Chicago, IL, USA) was used to compare the groups. The test was repeated by replacing samples between the groups until there were no significant differences (P=1.000). Then, the teeth were randomly distributed into six groups based on instrument brands (n=20). The sample size was estimated with a method similar to that used in previous studies (n=20) [10,11].

### Root canal cleaning and shaping:

An operator experienced in using full-sequence rotary and reciprocating systems prepared all the canals. The apical preparation size was set to #25 for each group, and all preparations were performed with a low-torque X-Smart plus endodontic motor (Dentsply Maillefer, Ballaigues, Switzerland). The teeth were irrigated with double-distilled water delivered through a side-vented needle (0.3×25mm, Endo-Top, Cerkamed, Stalowa Wola, Poland). The irrigation needle was inserted within 1 mm of the WL by using slight hand vibration and up-and-down motions. A total of 5mL of double-distilled water was used during each instrumentation and an additional 1mL of water was used for the final rinse. No glide path was created, because the initial canal sizes were equal to the size of a #15 K-file. Each single-file instrument was used to prepare only three canals, as was the ProTaper Universal, which included a set of files.

All instruments were used in accordance with their manufacturers' recommendations. The six instruments were as follows: Reciproc R25 (size 25, 0.08 taper, VDW, Munich, Germany), WaveOne Primary (size 25, 0.08 taper, Dentsply Maillefer, Ballaigues, Switzerland), OneShape (size 25, 0.06 taper, Micro-Mega, Besanco, France), F360 (size 25, 0.04 taper, Komet Brassler, Lemgo, Germany), Neoniti A1 (size 25, 0.08 taper, Neolix, Châtres-la-Forêt, France), and ProTaper Universal (SX, S1, S2, F1, F2, Dentsply Maillefer, Ballaigues, Switzerland). Each instrument was withdrawn from the canal after three in-and-out pecks. The flutes were then cleaned and inspected before being re-used. The canals were irrigated with double-distilled water, and a #10 K-file was used to confirm patency. This procedure was repeated until the file reached the WL.

### Collection of extruded debris:

The Myers and Montgomery method [10] was used in the present study. Empty vials without stoppers were weighed three times on different days with an electronic balance that had an accuracy of 10–5g (Precisa EP, Precisa Gravimetrics AG, Dietikon, Switzerland). The mean mass of each vial was determined and recorded. A hole was prepared in the stopper of each Eppendorf tube, and each tooth was fixed with glue up to the cementoenamel junction. Then, a vial stopper was perforated, and the Eppendorf tube’s cap was removed. The Eppendorf tube was inserted into the stopper of the vial, and it was suspended in a bigger vial. A second smaller vial was placed inside the bigger vial so that the end of the Eppendorf tube was exactly within the smaller vial. The bigger vial was used to protect the smaller vial (which was used to collect debris) from contamination. It also precluded the operator from seeing the teeth and the amount of extruded irrigant during canal preparation.

A 27-gauge needle was placed alongside the stopper of the bigger vial to balance the air pressure inside and outside the tube. Gaps between the stopper, the needle and the Eppendorf tube were sealed with adhesive to prevent extruded irrigant from leaking through these gaps into the vial. After the instrumentation of each sample, the tooth and the stopper of the vial containing the Eppendorf tube were removed, and the tooth was washed with 1mL of double-distilled water in the vial to collect debris that was attached to the root surface. To evaporate the double-distilled water, the vials were incubated at 70°C for five days. The final mass of the dried debris was obtained by weighing the samples three times and recording the mean mass. The net mass of the extruded debris was calculated by subtracting the initial mass (the mass of the empty vial) from the final mass.

### Preparation time:

The total preparation time for each system was recorded and included the time required for active instrumentation, cleaning the file flutes, changing the instruments and irrigation.

### Statistical analysis:

The normality of the data was confirmed using the Kolmogorov-Smirnov test, and data were analyzed using one-way ANOVA. The Levene's test showed heterogeneity of variances; therefore, Game-Howell post hoc test was used at 95% confidence interval (P=0.05).

## RESULTS

Table 1 shows the mean mass and standard deviation of the apical debris produced by each rotary system. The ProTaper Universal produced the highest amount of debris, and the F360 produced the least amount of debris. There was a significant difference between the ProTaper Universal and the F360 (P=0.02). Pairwise comparisons of the other systems showed no significant differences (P>0.05).

**Table 1:** Amount of apically extruded debris (milligrams) and preparation time (seconds) by each rotary system (n=20)

** Type of Instrument **		** Reciproc **	** WaveOne **	** OneShape **	** F360 **	** Neoniti A1 **	** ProTaper Universal **
** Debris **	** Mean **	.97614	1.10983	1.29466	.79700^*^	1.70233	2.07117^*^
** Extrusion **	** SD **	.47768	.67648	.83966	.53143	1.30607	1.38012
** Preparation **	** Mean **	42.85 ^ a,b ^	54.85 ^ a,c,d ^	39.68 ^ a,b ^	32.74 ^ b ^	61.65 ^ c ^	88.60 ^ d ^
** time **	** SD **	10.94	25.97	20.42	2.88	4.19	10.73

SD: Standard deviation

^*^Indicates a significant difference in the amount of extruded debris in milligrams (P<0.05)

Different superscripted letters indicate a significant difference between groups in preparation times (P<0.05)

Canal preparation took significantly longer with the ProTaper Universal than with the F360 (P=0.0001). In addition, the total preparation time was significantly longer with the Neoniti A1 than with the Reciproc R25 (P=0.008), OneShape (P=0.006) and F360 (P≤0.0001). Furthermore, the total preparation time with the WaveOne Primary was significantly longer than with the F360 (P=0.03). Table 1 shows the mean preparation time for each rotary system.

## DISCUSSION

The present study quantified the amount of extruded apical debris and compared five single-file rotary systems to the conventional, well-studied ProTaper Universal rotary system in this regard. The results of the present study showed that the highest and the lowest amounts of apically extruded debris were produced by the ProTaper Universal and the F360, respectively. Additionally, significant differences were only found when the ProTaper Universal was compared to the F360 (P=0.02). No significant differences were found between the other systems.

Ozsu et al, [12] in 2014 showed that the WaveOne Primary extruded less debris than the ProTaper Universal, which is consistent with our result. In another study, the ReciprocR25 was compared with a self-adjusting file (ReDent Nova, Ra'anana, Israel) and the ProTaper Universal and Revo-S (Micro-Mega, Besançon, France) systems; no significant differences were found between the systems in this respect [13]. However, the ReciprocR25 did result in the least amount of extruded debris, and the ProTaper Universal resulted in the highest amount of extruded debris [13]. Nevertheless, when the single-file reciprocating WaveOne and Reciproc instruments were compared to full-sequence rotary instrumentation systems (ProTaper Universal and Mtwo), the single-file systems caused more debris than the other systems [14]. Another study compared three single-file systems (Reciproc, F360, and OneShape) with the Mtwo full rotational multi-file system and found that the Reciproc extruded more debris than the other systems [15]. These conflicting results could be due to various sizes and tapers of the files that were compared. In another recent study, Silva et al, [16] in 2015 compared WaveOne (large files size 40, 0.08 taper), Reciproc (R40 size 40, 0.06 taper), ProTaper Next (last file X4 size 40, 0.06 taper) and ProTaper Universal (last file F4 size 40, 0.06 taper) and reported that the ProTaper Universal resulted in extrusion of more apical debris than the other systems [16]. Preparing the entire canal with only one single file instead of sequential multi-file systems has simplified instrumentation and could be one of the reasons that single-file rotary systems result in less extrusion of debris [17]. However, in the present study, this speculation was not supported because the ProTaper Universal produced similar amounts of extruded debris compared to the WaveOne Primary, OneShape, and Neoniti A1. It has been suggested that file kinematics, tapers, and designs could result in more apically extruded debris, but there is not enough evidence to prove these relationships [15]. In the present study, the F360 resulted in less extrusion of debris than the other systems, but the difference was significant only with respect to the amount of debris produced by the ProTaper Universal. One reason could be that the F360 file taper (0.04) is smaller than the tapers of other systems. The cross-sectional designs of the F360 and the Reciproc R25 are similar; both systems are S-shaped and can facilitate the movement of debris in coronal direction, but they differ in taper and rotational motion. The WaveOne Primary and ProTaper Universal feature a modified triangular cross-section, which results in lower cutting efficiency and smaller chip space [18]. The OneShape has a different cross-sectional design along the entire WL of its file. According to the manufacturer, the Neoniti A1 has Gothic-like tip design and builtin abrasive properties. Since there were no significant differences in the present study between single-files with different rotational motions, designs, and tapers, more evidence is required to determine the effects of these factors on apical extrusion.

It is unclear whether the differences between the systems can be extrapolated to clinical situations, as the amount of extruded debris could be harmless or similarly deleterious for periapical tissues. The type of roots that were chosen for this study could be one of the reasons that the debris levels were low. Single wide canals of human mandibular premolars could limit the pumping effect of the file during insertion and consequently result in less apical extrusion of debris [19]. In this regard, narrow canals without coronal flaring could extrude more debris [20]. Adjusting the WL at 1mm distance from the apical terminus could reduce the amount of extruded debris [5]. Another reason for low amounts of apically extruded debris could be the use of a side-vented irrigation syringe. A longer preparation time is required when using the ProTaper Universal because several files are being used to complete canal preparation. The shorter preparation time of single-file systems seems attractive; but in the present study, the Neoniti A1 required more time than the F360 for canal preparation; one possible reason could be abrasive cutting edges of the file and its cutting efficiency. Further studies are required to compare cutting efficiencies of different single-file systems.

## CONCLUSION

In this study, the F360 single-file rotary system extruded less debris than did the multi-file ProTaper Universal, although all the tested instrumentation systems did extrude debris apically.
